# Vertical organization of the division of labor within nests of the Florida harvester ant, *Pogonomyrmex badius*

**DOI:** 10.1371/journal.pone.0188630

**Published:** 2017-11-28

**Authors:** Walter R. Tschinkel, Nicholas Hanley

**Affiliations:** Department of Biological Science, Florida State University, Tallahassee, Florida, United States of America; Montana State University Bozeman, UNITED STATES

## Abstract

In the Florida harvester ant, *Pogonomyrmex badius*, foragers occur only in the top 15 cm of the nest, whereas brood and brood-care workers reside mostly in the deepest regions, yet the food and seeds foragers collect must be transported downward 30 to 80 cm to seed chambers and up to 2 m to brood chambers. Using mark-recapture techniques with fluorescent printer's ink, we identified a class of workers that ranges widely within the vertical structure of the nest, rapidly moving materials dropped by foragers in the upper regions downward, and excavated soil from deeper upward. Within the nest, only 5% of foragers were recovered below 20 cm depth, but about 30% of transfer workers and 82% of unmarked workers were found there. Below 70 cm depth, 90% of workers were unmarked, and were probably involved mostly in brood care. During the summer, the transfer workers comprise about a quarter of the nest population, while foragers make up about 40%. Workers marked as transfer workers later appear as foragers, while those marked as foragers die and disappear from the foraging population, suggesting that transfer workers are younger, and age into foraging. The importance of these findings for laboratory studies of division of labor are discussed. The efficient allocation of labor is a key component of superorganismal fitness.

## Introduction

Division of labor is a hallmark of insect sociality, and is expressed along multiple axes. In social hymenoptera, the defining division is between reproductive (queen) and non-reproductive (worker) individuals, but specialization within each of these castes also occurs. Within the worker caste, division of labor is almost universally associated with worker age (age polyethism), and in those species with highly size-variable workers, with body size. Division of labor has been reviewed a number of times [1,2,3,4] and subjected to several theoretical or modeling treatments [1, 5–7]. In age polyethism, young workers are associated with brood care, moving to more general nest tasks as they age, and becoming foragers outside the nest in old age. These functional transitions have been described in dozens of species of ants (for more recent examples: [5,6]). In addition to changes in behavior, age polyethism is associated with a number of physiological, developmental, sensory and genetic changes. Gene activity changes [7], as do hormone levels [8], ovarian development [9,10], lipid stores [11] and responsiveness to task stimuli [12,13]. Occasional examples of the absence of age polyethism also exist [14,15]. A perennial question has been the degree to which division of labor and task selection are flexible or causally associated with age [7,8]

Unfortunately, most division of labor studies in ants have been carried out in artificial, simple laboratory nests that bear little or no resemblance to natural nests, or to natural ecological conditions. Laboratory studies have therefore usually missed two important aspects of division of labor. First, with a few exceptions (see below), laboratory studies are insensitive to the spatial organization of division of labor, that is, that shifting worker roles depend upon movement among particular parts of natural nests. And second, in nature, age-related transitions between worker roles are regulated by mortality of foragers, but such mortality is artificially lower in the laboratory. These two issues are described below.

### Division of labor is organized within nest space

Porter and Jorgensen [16] and MacKay [17] showed that within their nests, several species of harvester ants are vertically stratified by age, with young workers and brood deep in the nest, and foragers and defenders in the upper regions. Tschinkel reported a similar distribution in *P*. *badius* [18] and in *Prenolepis imparis* [19]. In all of these, the young workers deepest in the nest had the highest fat content and dry weight, and the lowest respiratory rate, whereas the foragers in the upper regions had the lowest fat content [16,17,18,19,20,21] creating additional correlations. These studies suggest that as workers age, they move upward and change behavioral and physiological roles, finally foraging outside the nest as the last act of their lives. This adds a physical dimension to the age-related changes in worker behavior and physiology.

Workers of some roles seem to be more strongly segregated than others. Most obvious are foragers, as they are always found in the upper nest region [16,17]. The sharpness of this limitation was revealed through mark-recapture in *P*. *badius* by Kwapich and Tschinkel who found that foragers were completely limited to the top 15 cm of the nest [22,23]. A similar segregation of workers was found between fungus garden workers and trash workers in the leafcutter ant, *Atta cephalotes* [24], a segregation that reduces the introduction of pathogens into the nest [25].

In contrast to the majority of age polyethism studies, a few laboratory studies included a reasonably realistic spatial component. For the tiny colonies of *Leptothorax albipennis* and *L*. *unifasciatus* a laboratory nest between plates of glass may not be radically different from their natural nest in the thin spaces between stones. In such nests, workers of various roles are segregated in space, and resume that organization after forced re-nesting [26]. Other laboratory studies also showed spatial fidelity associated with division of labor [27,28]. The latter but not the former tested the effect of worker age, but neither revealed the degree to which the laboratory nest resembled the natural nest.

### Extrinsic forager mortality shapes division of labor

Kwapich and Tschinkel found that most forager mortality resulted from extrinsic causes, and that whereas forager lifespan was only about 3 weeks in nature, it was many months in the laboratory [23]. In other words, in nature, foragers do not die of old age. Evolution over many generations has thus tuned colony demography to replace foragers at a rate determined by the average extrinsic mortality rate. An experimentally increased loss of foragers was not met with replacement beyond the demographic rate, but reduced forager mortality (by penning focal or neighboring colonies) inhibited the transition of younger workers into the foraging class. This implies that age-related division of labor is driven, in part, by extrinsic forager mortality, a factor that is almost absent in the laboratory. Whether transitions between other worker roles are also affected by forager mortality rate is unknown.

This paper is not so much about age polyethism as it is about identifying the component parts of the superorganism and how they are organized to carry out the various necessary functions. Because foragers never venture far below the nest surface, and the brood and their caretakers reside overwhelmingly in the deepest nest regions, there should exist an intermediary worker class to transport food and other material between these non-contacting groups. We present evidence that such a distinct class of transfer workers operates in the Florida harvester ant, *P*. *badius*, and that its operation is associated with worker age and location. We also argue that its transition to foraging is controlled by forager mortality.

## Materials and methods

### Study site

The study population of Florida harvester ant, *Pogonomyrmex badius*, is located in a 23 ha site (latitude 30.3587, longitude 284.4177) about 16 km southwest of Tallahassee, Florida, USA, within the sandhills portion of the Apalachicola National Forest. The site, Ant Heaven, consists of excessively drained sandy soil occupying a slope to a wetland and stream, causing its water table to be depressed (>5 m at the maximum), thereby making it suitable for several drought-resistant species of plants and animals. The forest consists of longleaf pines (*Pinus palustris*), turkey oak (*Quercus laevis*), bluejack oak (*Quercus incana*) and occasional sand pines (*Pinus clausa*) and sand live oak (*Quercus geminata*). The natural ground cover consists of several successional grasses and forbs, along with clonal shrubs such as shiny blueberry (*Gaylussacia dumosa*) and gopher apple (*Licania michauxii*).

This study was carried out under US Forest Service, Apalachicola National Forest permits APA583 and APA56302. No protected species were involved.

### Fluorescent marking procedure

**Fig 1:**
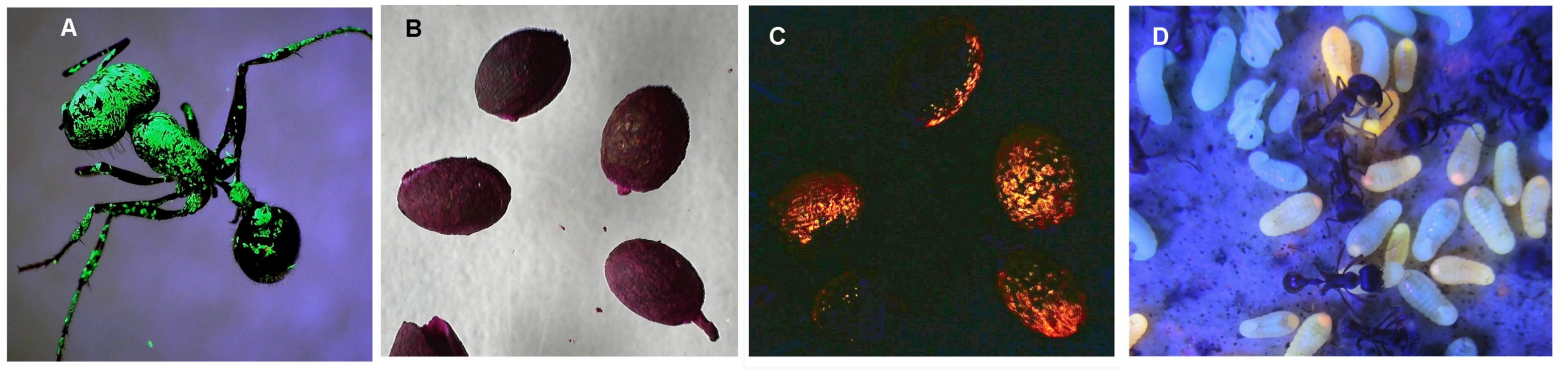
Marking with fluorescent printers ink and rhodamine dye. A. a forager under UV light; B. seeds under visible light; C. the same seeds under ultraviolet light; D. ant larvae fluorescing after consuming rhodamine B-dyed food.

Marking objects and ants with fluorescent inks of different colors in order to recapture and distinguish them later was a central technique of this study. Using fluorescent printers ink dissolved in ether and sprayed on objects as a fine mist was first reported by Porter [29]), and was later adopted and further developed by Tschinkel [30] and Kwapich and Tschinkel [22] for mark-recapture estimation of ant forager populations. Printers ink in several fluorescent colors (green and orange were most commonly used) was purchased from Gans Co. (http://www.gansink.com/locations.asp) and diluted 1 part ink in 9 parts diethyl ether. This was sprayed on ants or seeds in a tray using a 5 mL, plastic perfume mister (Freund Container and Supply, freundcontainer.com), and allowed a few minutes to dry. Most colors were not easily seen in visible light, but shone like beacons under ultraviolet (Fig 1). There was no effect on ant mortality or behavior, and the ants handled marked seeds like unmarked seeds.

The fluorescent, vital, water-soluble dye, rhodamine B was used to dye pieces of tenebrionid beetle larvae (*Zophabas atratus*) which, when offered to *P*. *badius* colonies, were fed to ant larvae, causing them to fluoresce brightly under UV light (Fig 1D). This allowed the detection of the transport and ingestion of the pieces of beetle larvae.

### Fluorescent marking of foragers

Each trial began with exhaustive marking of foragers [22]. Foragers were collected by baiting with seeds and cookie crumbs at least 1 m from the nest entrance. Foragers were defined as those workers that picked up bait and headed back to the nest. These foragers were captured and spray-marked with green fluorescent printers ink dissolved in ether (see above). Using the same color ink, this was repeated over several days, until 80% to 90% of foragers coming to baits were marked (one nest was only 75% marked).

Kwapich and Tschinkel [22] reported that fluorescent-marked foragers were found only in the top 15 cm of the nest, but that even when almost all foragers coming to baits were marked, excavation of the top 15 cm revealed that 54% of workers there were unmarked (i.e. were not foragers). It thus seemed likely that these unmarked workers in the upper parts of the nest potentially functioned as "transfer workers " (hereafter referred to as such) that traveled up and down in the nest moving seeds, food, soil and brood. The focus of this paper is on the identity and movement of these possible transfer workers. An additional hypothesis is that these unmarked workers are soon to become foragers, but the two hypotheses are mutually compatible.

### Fluorescent marking of transfer workers

While foragers can be marked without disturbing the nest, transfer workers cannot, yet if the movements of marked transfer workers in the nest are to be studied, the upper zones of the nest disturbed during the capture of transfer worker must be restored to a reasonable facsimile of their original state. This was done as follows, once most of the foragers had been marked.

One day after forager marking was complete, the top 15 cm of the nest was excavated by sequentially exposing chambers with a brick trowel and lifting off chamber ceilings (as described in [18,22]). After collecting all workers from the exposed chambers, they were inspected under a UV light in order to separate green-marked foragers from unmarked workers. The unmarked workers were marked with orange fluorescent ink. Most of these "non-foragers" were what we provisionally designated as transfer workers, but also included the 9% to 20% of foragers that remained unmarked. These were then not distinguishable from transfer workers later in the study. We did not expect to recover all marked foragers in the top 15 cm because the nest was open and some foragers were afield.

### Reconstruction of the nest

**Fig 2:**
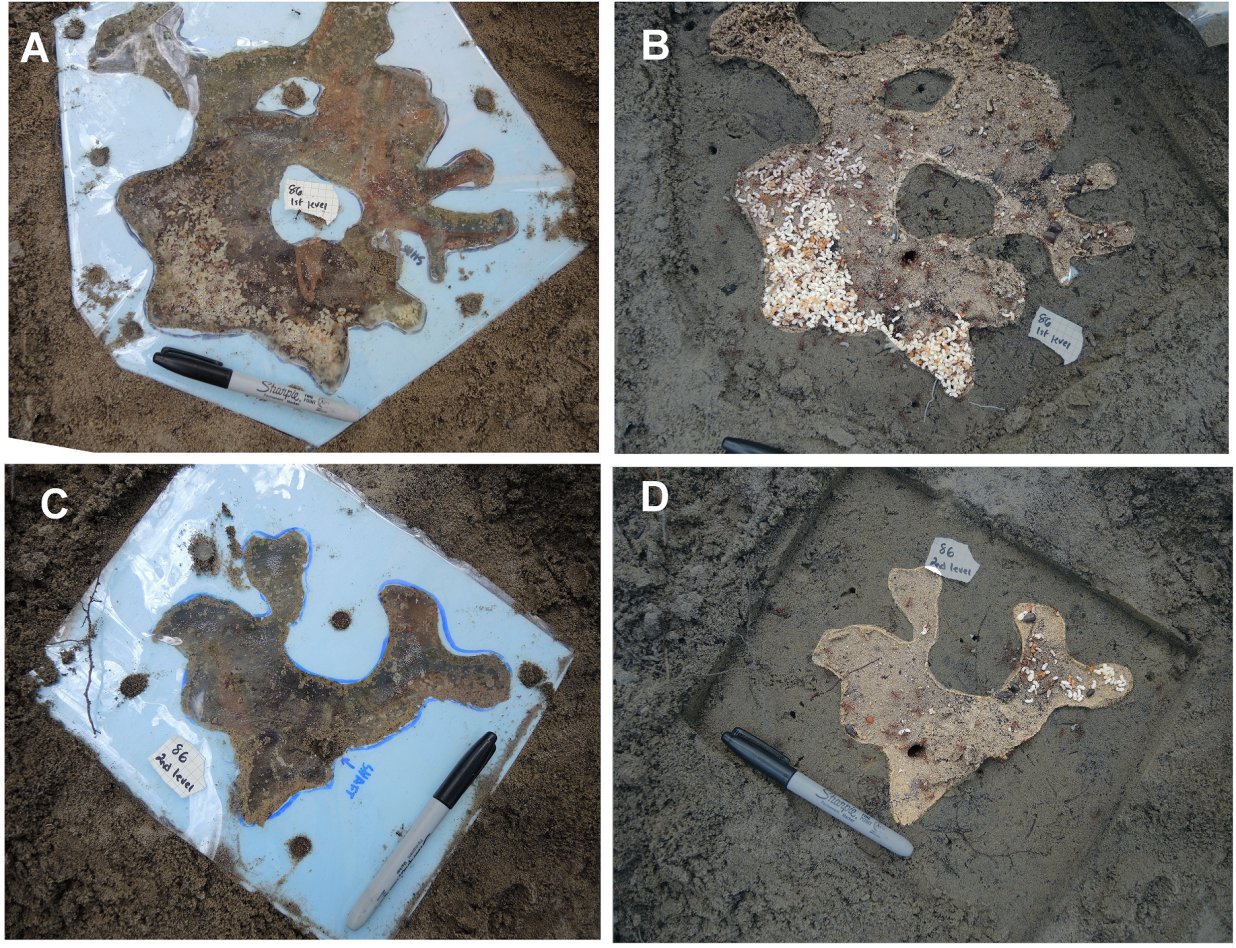
Examples of chamber facsimiles and their use by the ants in the reconstructed nests. The foam board has been removed in the right panels, showing chamber contents. The non-chamber area in the right images has been digitally darkened to increase the visibility of the occupied chamber area. Note the brood (mostly pupae) in the right panels, and the sand from deeper levels deposited in the lower right chamber. Although brood can sometimes be found in the warmer, near-surface chambers, they compose only a small fraction (<2%) of a nest's total brood; data from [32].

When each chamber had been exposed and emptied, a piece of transparent acetate film was placed over it and its outline traced. These outlines were transferred to a sheet of 1.3 cm thick foam insulation panel and then cut out to create a facsimile of the chamber. After adding a ceiling of acetate film held on with nails, the chamber-facsimile was buried at the same depth as the original chamber, covered with sand and provided with a connecting shaft to the chambers below and above. In this way, two to four real chambers were replaced with facsimiles of the same area and outline (Fig 2).

### Nest excavation and evaluation

Upon completion of the reconstructed nest, all workers, brood and other captured contents were released in an enclosure surrounding the nest entrance. The enclosure was removed in 24 hr or less. Two to six days later, colonies were offered fluorescent-marked seeds and tenebrionid larva pieces dyed with rhodamine B. Earlier work [22] had shown that such food items are quickly retrieved into the nest and transported downward, presumably by a class of workers other than foragers, that moves up and down in the nest. One to three hours after baiting, the vertical distribution of marked foragers and transfer workers within the nest was determined through chamber by chamber excavation and capture, following the procedures of Tschinkel [18] and Kwapich and Tschinkel [18,22]. Contents of each chamber were kept separate and were checked for fluorescent marks under UV in the laboratory. Fluorescent items scored included green-marked foragers, orange-marked transfer workers, seeds, pieces of dyed beetle larvae, fluorescing larvae and callow workers that had eaten the dyed tenebrionid larvae (Fig 1D). This procedure was applied to 11 colonies during the active season in 2016 and 2017.

### Post-excavation procedure

After census and evaluation of nest contents in the laboratory, we created a subterranean nest in the colony's original location using ice [31], and released the colony into this nest after the ice melted. All colonies readily moved into these nests. At various elapsed times up to 50 days, foragers were captured as above and checked for green and orange-marked workers in order to evaluate forager survival and turnover.

### Data analysis and availability

Data were graphed and analyzed by ANOVA, t-test and regression using Statistica 13 (Statsoft Inc.). Data are available in S1 Table and S2 Table.

## Results

### Use of facsimile chambers

All colonies used the facsimile chambers that replaced the destroyed chambers in the top 15 cm of their nests (Fig 2). The contents and activity of the facsimile chambers were similar to those of natural nests [18] [22]. In several cases, sand excavated from lower in the nest was deposited on the floors of the facsimile chambers (Fig 2B and 2D), as it often is in natural nests as well [18]. Relatively normal use of the facsimile chambers is a necessary condition for the subsequent assessment of marked-worker distribution within the nest.

### Recovery of marked workers

Forager marking proceeded for a variable number of days. In eight experimental colonies, 80% to 91% of foragers were marked in three to five days, but two colonies required non-sequential marking efforts for 11 to 12 days to reach this criterion, and one 45 non-sequential days. The total number marked was referred to as the cumulative number of marked foragers. One day after forager marking was complete, the top 15 cm of the nest was excavated and all unmarked workers were marked as transfer workers (orange). All workers were then returned to the reconstructed nest. Because colonies were actively foraging during this procedure, only about 54% (s.d. 18%) of the cumulative marked foragers were recaptured in the top 15 cm. When adjusted for a mean of 14% of foragers that were unmarked, this value was about 62%.

Two to six days were allowed for the colony to occupy and adjust to the facsimile chambers. Just before excavation, colonies were offered seeds and dyed beetle larvae. One to three hours later, all workers on the surface were collected, followed by excavation and collection of all nest contents, chamber by chamber. Overall, 71% (s.d. 23%) of the marked transfer workers were recovered in the final nest excavation, but only 39% (s.d. 19%) of foragers were recovered. Lower forager recovery resulted from two factors—many were probably afield when the nest was excavated, and about 4% per day had died while foraging [22,23]. Given two to six days elapsing between completion of marking and final nest excavation, this amounted to a loss of 8% to 22% of foragers, respectively. Similar losses were also incurred during the more extended marking periods, but were difficult to estimate. There was a trend for lower forager and transfer worker recovery with longer elapsed times, but this was not quite significant. In any case, the central question of this paper does not require recovery of all marked workers because the measure of interest is their relative distribution within the nest.

### Recapture on the nest surface

**Fig 3:**
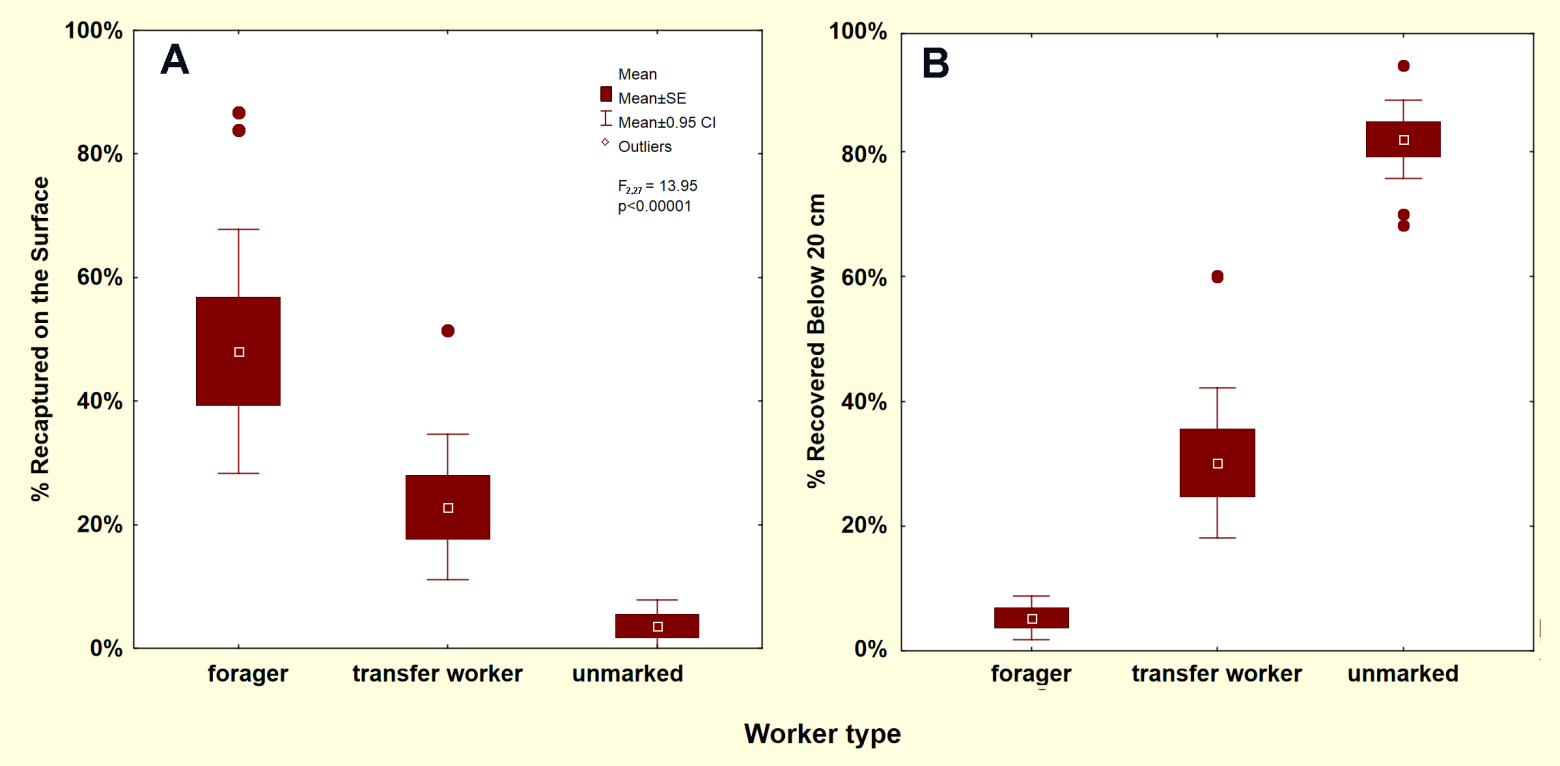
**Capture of workers on the nest surface (A), and deeper than 20 cm in the nest (B).** Marked foragers were captured primarily on the ground surface and were essentially absent below 20 cm depth. Transfer workers occurred in both regions, and unmarked workers primarily deep in the nest.

Because foragers are by definition active outside the nest, and because baiting and excavation were necessarily carried out while the colony was actively foraging, a large proportion of the total foragers recovered during final excavation were captured on the surface (Fig 3A). Fewer than half as many transfer workers and almost no unmarked workers were captured there (Fig 3A; F _2,27_ = 13.95; p<0.00001). A similar proportion of transfer workers occurred below 20 cm (Fig 3B). In contrast, foragers were essentially absent below 20 cm (Fig 3B), a region in which most workers were unmarked.

### Vertical distribution of workers within the nest

Of greatest interest for this study is how the marked and unmarked workers recovered *underground* distributed themselves in the vertical space of the subterranean nest. All foragers were originally captured and marked on the surface, and all transfer workers were originally captured and marked in the top 15 cm of the nest. Does either class move up and down in the nest with significant frequency? If so, they would fulfill one of the requirements for transfer workers. Because colonies differed greatly in population size, the vertical distribution of these worker classes was expressed as percent of each group in each 10 cm increment of nest depth.

**Fig 4:**
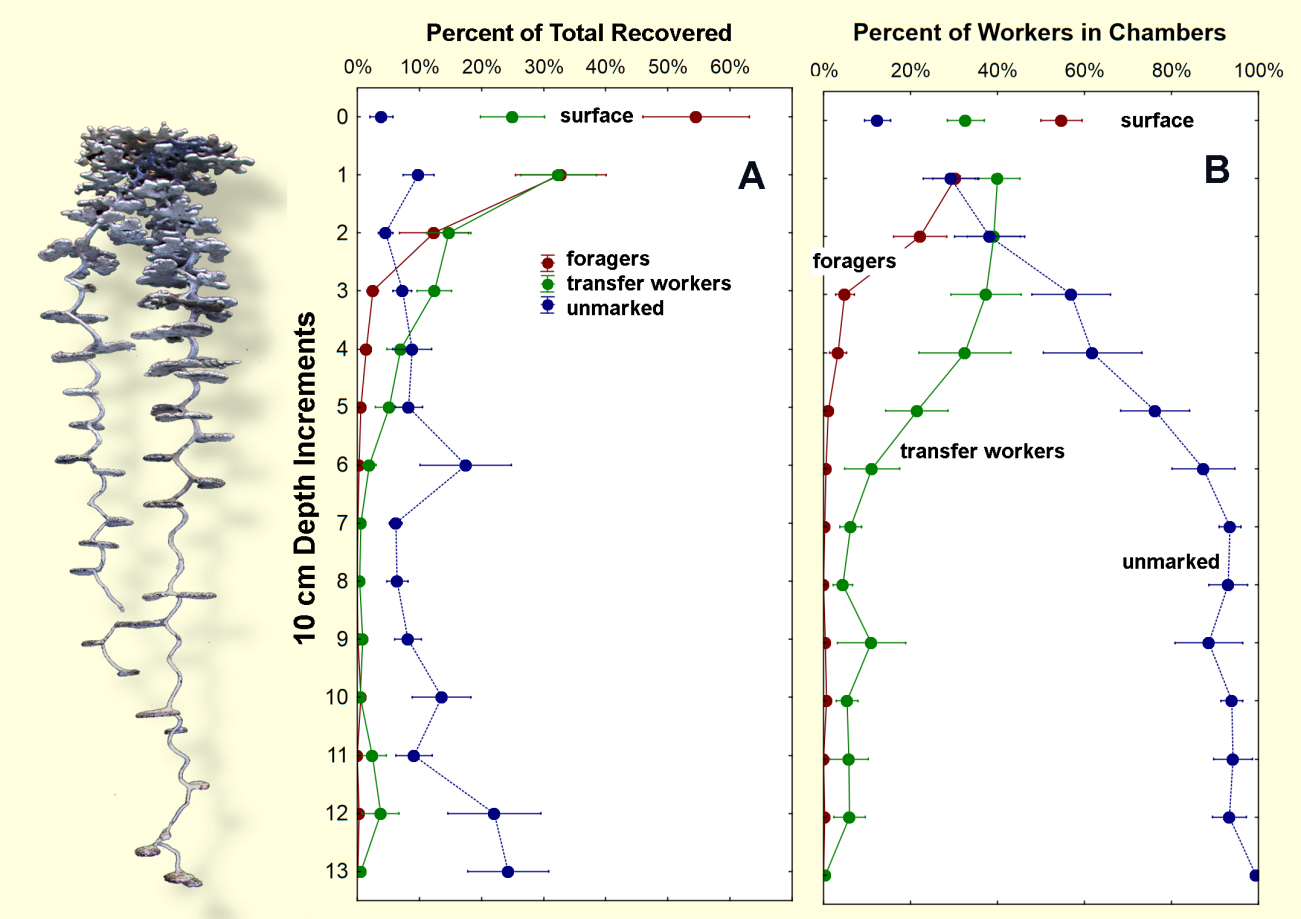
Distribution of marked and unmarked workers within the nest. Depth is shown in 10 cm increments. A. percent of the total of each type recovered; B. percent of each type chamber by chamber. Photo on the left is an aluminum cast of an approximately average *P*. *badius* nest.

Surface capture is distinguished from forager baiting by taking place on the mound surface without baits. Fig 4A shows that about 95% (s.e. 1.6%) of ants marked as foragers were recovered either on the surface (55%) or in the top 20 cm of the nest (40%). A mean of only 5% (s.e. 1.6%) of foragers occurred below 20 cm. In other words, foragers were predominately recaptured in the zone in which they were captured in the first place. On the other hand, marked non-foragers (presumed transfer workers) first captured in the top 15 cm of the nest did not remain there, but were spread much more evenly across the nest's vertical structure. About 30% (s.e. 5.4%) were recovered below 20 cm, with some as deep as 110 and 120 cm, the depth at which larvae normally reside. The recovery of a large number of transfer workers on the surface (about 25%) suggests several possibilities: (1) they were foragers that were mismarked as transfer workers (about 14% of foragers), (2) they were sand workers whose role was to take out excavated sand, trash, etc. These are intermediate in age, and later transition into foragers[33]; (3) they were transfer workers transitioning directly into foragers. These transitions remain to be well-characterized.

Unmarked workers increased steadily with depth, with 82% (s.e. 2.8%) occurring deeper than 20 cm (Fig 4A). Below 70 cm, they made up over 90% of the workers.

Because the subterranean distribution of transfer workers is the primary focus of this study, Fig 4B shows the chamber-by-chamber ratios of the three worker classes. Whereas foragers were essentially absent from chambers deeper than 20 cm, transfer workers were found below that depth, and continued to make up a significant proportion of the workers in chambers. Below about 70 cm, transfer workers made up a mean of about 6% (range 0.04% to 10.3%) of the workers, with unmarked workers making up the difference (mean 94%). The occurrence of workers that had been marked in the top 15 cm at such nest depths suggests that *transfer workers*, *in contrast to foragers and unmarked workers*, *are vertically mobile*.

### Downward transport of food

**Fig 5:**
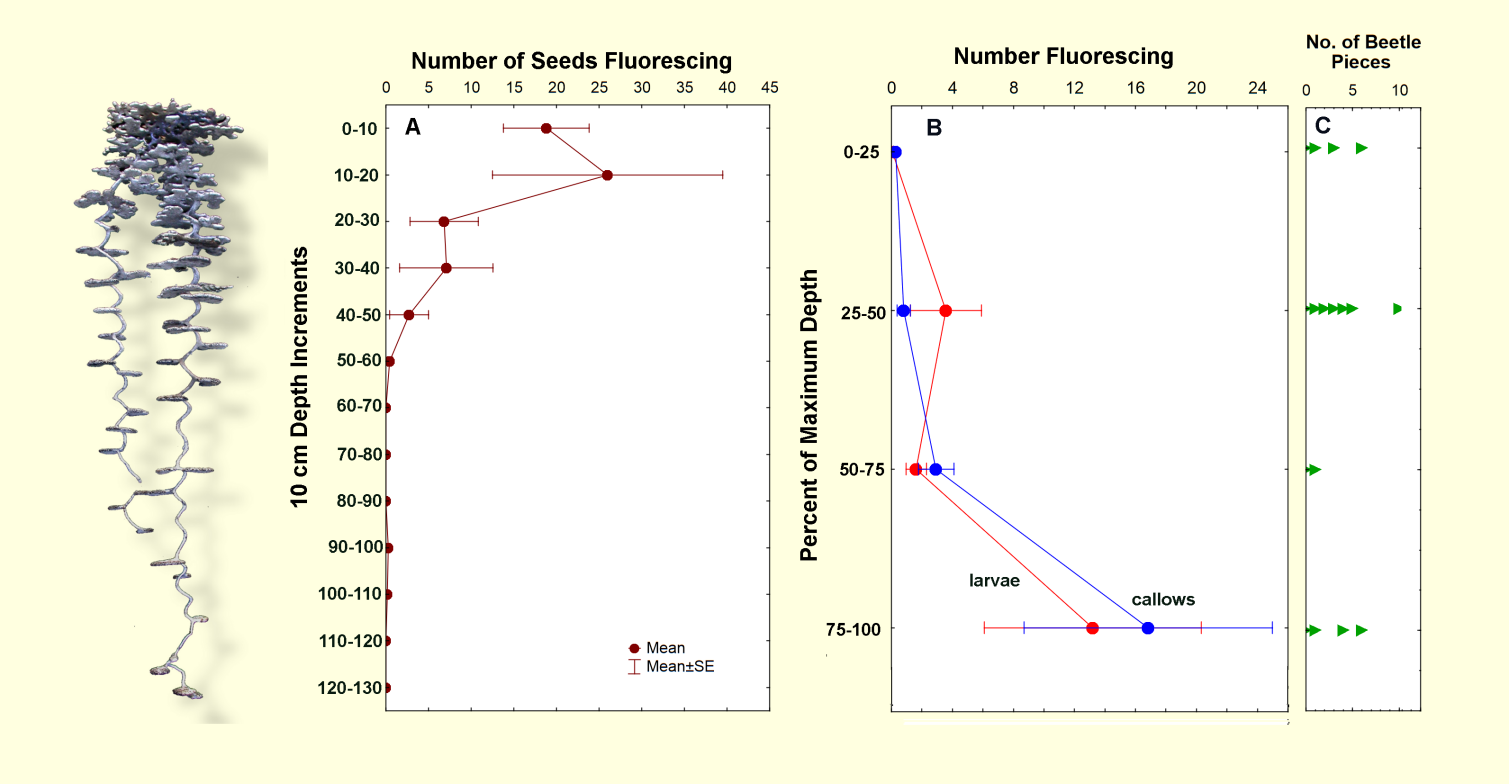
Distribution of marked seeds, larvae, callow and beetle pieces by depth. A. Distribution of marked seeds in 10 cm increments of depth; B. Distribution of fluorescing larvae and callows in quarters of maximum nest depth. Fluorescence resulted from feeding on dyed beetle pieces; C. Distribution of dyed beetle pieces in quarters of maximum nest depth. Eight nests were offered beetle pieces, and 2 to 30 pieces were recovered in the nest. Photo on the left is an aluminum cast of an approximately average *P*. *badius* nest.

The apparent vertical mobility of transfer workers is supported by the rapid movement of seeds and beetle larva pieces deep into the nest, a movement unlikely to be carried out by foragers because these are essentially absent below 20 cm. During the one to three hours elapsing between the offering of fluorescent-marked seeds and dyed beetle pieces, foragers collected these items and deposited them in the top 15 cm of the nest [26]. During the subsequent nest excavation, these items were found at all levels of the nest, far deeper than their original locale of deposition (Fig 5). The total marked seeds brought into the nest ranged from 17 to 294, and of dyed tenebrionid pieces, 2 to 30. The presence of between 5 to 258 fluorescing ant larvae, and 14 to 180 fluorescing callow workers showed that the dyed pieces had actually been eaten. Two colonies even contained 6 and 7 fluorescing pupae.

**Fig 6:**
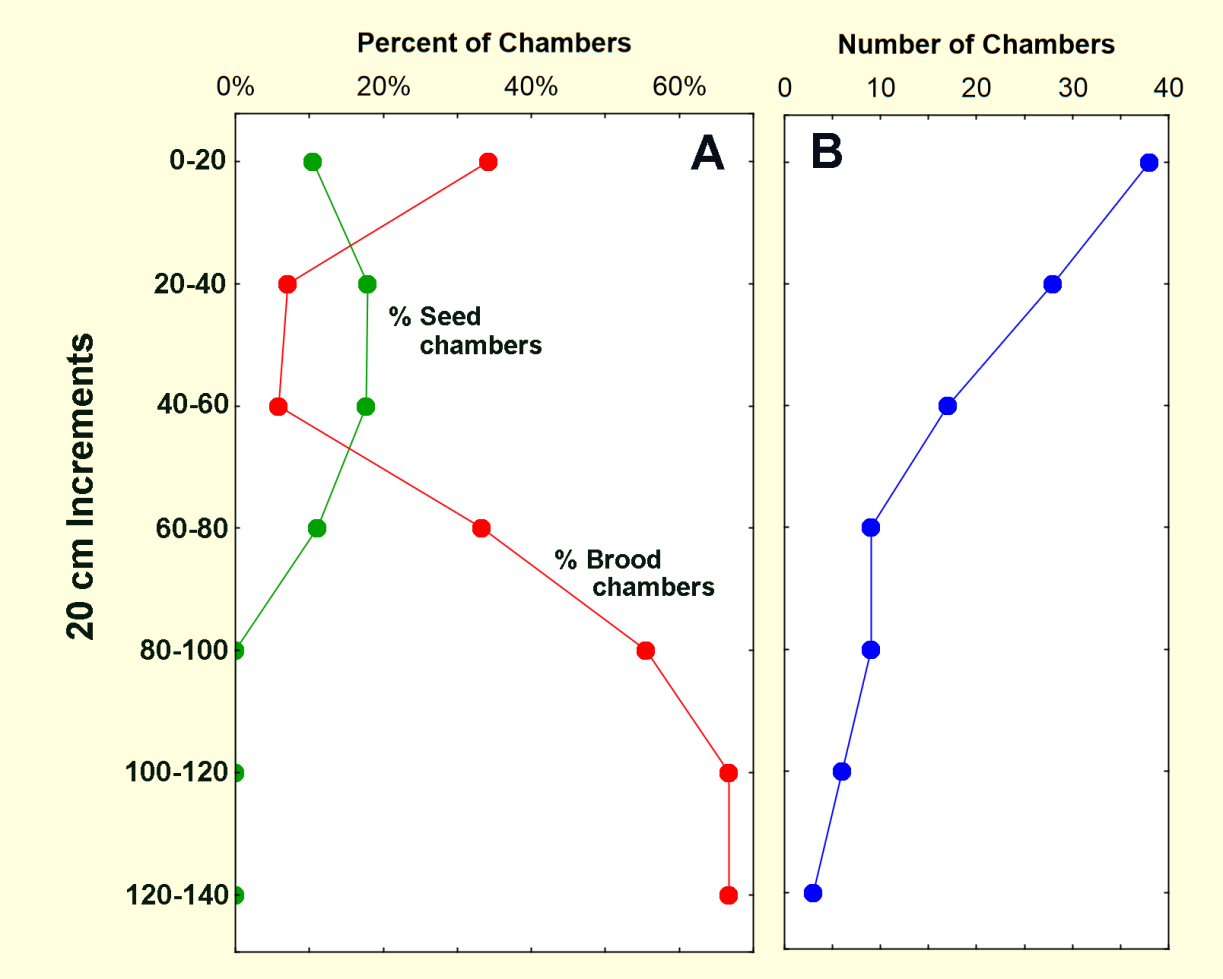
Distribution of chambers by type and nest depth. A. Seed and brood chambers as a percent of the total; B. Total chambers.

Fluorescing seeds were most abundant in the top 20 cm of the nest, reflecting their rapid deposition in this zone by foragers (Fig 5A). No marked seeds made it deeper than about 60 cm reflecting the depth of most seed storage chambers (between 30 and 80 cm) (Fig 6A). This is also the zone in which transfer workers made up 20% to 40% of workers in chambers, whereas at depths greater than 50 cm, they made up 0 to 10%. This pattern suggested that a large proportion of transfer workers moved seeds into storage chambers, as seems reasonable for a seed-harvesting ant. Fewer serviced the brood chambers located at greater depth than seed chambers (>80 cm) (Fig 6A).

In contrast to seeds, fluorescing larvae and callows were most abundant in the deepest regions where 98% of brood are normally kept [34] (Fig 4B; Fig 5B; Fig 6A). The fluorescence was derived from feeding on dyed beetle-larva pieces, the majority of which were recovered between 0 and 60 cm, with about 12% deeper (Fig 5C). Not all dyed pieces had been eaten, but clearly enough had to cause a large number of ant larvae and callows to fluoresce.

### Colony composition by worker roles

What fraction of the colony performs each of the three major roles, forager, transfer worker and unmarked (probably younger) worker? These values were approximated as follows: (1) the forager population was estimated from the cumulative number marked and the final percent marked, discounted for forager mortality of 4% per day for the days elapsing between the last mark day and final nest excavation. This discount ranged from 8% to 22%; (2) the estimated unmarked foragers were added to the forager estimates; (3) transfer workers marked during the excavation of the top 15 cm were counted directly; (4) the unmarked foragers were subtracted from the transfer worker counts because unmarked foragers were almost certain to be in the top 15 cm of the nest. They were, of course, mis-marked with the transfer worker color (orange).

These estimates yielded the total ants in each colony and the percent of workers in each role. Colonies ranged from about 500 to 4500 workers (mean 2000, s.d. 1250). Foragers averaged 41% (s.d. 6%) of the colony, transfer workers 23% (s.d. 13%) and unmarked 36% (s.d. 14%). Transfer workers are thus a minority of the workers, but together with unmarked workers make up almost 60% of the colony. Our mid-summer estimate of the percent foraging is somewhat higher than the more precise mid-summer peak of about 35% reported by Kwapich and Tschinkel [22], but unlike theirs, ours was independent of colony size (p> 0.5; n.s.). On the other hand, the fraction of transfer workers decreased with colony size (fraction transfers = 0.35–0.000059* total workers; p<0.08; R^2^ = 31%) while the fraction of unmarked workers increased at a similar rate (fraction unmarked = 0.23+0.000068* total workers; p< 0.05; R^2^ = 36%).

### Post-excavation turnover in marked foragers

If transfer workers are ageing into foragers, and foragers are dying on the job, then ants bearing the forager mark (green) should gradually fade from the foraging population while ants bearing the transfer worker mark (orange) should increase, as should unmarked workers. This is just what happened when foragers were assessed 20 or 50 days after the colony was replanted in its original location. The proportion bearing the green forager mark decreased from about 79% (s.d. 15%) on the final day of forager marking to 12% (s.d. 6) after 20 days and 7% after 50, days. At 50 days, three of six colonies had no green-marked foragers at all. At the same time, the proportion bearing the orange transfer worker mark increased from 0% at the time of initial marking to 41% (s.d. 13%) at 20 days and 42% at 50. At the same time, unmarked foragers increased from about 14% (s.d. 6.1%) to 46% and 52%.

## Discussion

Division of labor and worker age in the Florida harvester ant are both strongly associated with location within the vertical nest structure, suggesting that there is an essential spatial element to division of labor. Foragers occur only in the uppermost chambers of the nest, where they deposit items brought in from the field [16,22], while the deepest regions of the nest are home mostly to brood and young workers who act as nurses for the brood [34], although some brood may be found temporarily in the warmer upper nest regions. There seems to be little or no direct exchange between these two groups, as neither normally ventures far from their "home" zone. We have now shown that a third class of workers acts as shuttles, transferring items of food downward and excavated soil (and possibly brood under some circumstances) upward. These non-foraging, vertically-mobile workers have several of the characteristics expected of such shuttle or transfer workers—(1) they can be captured in the upper chambers where their distribution completely overlaps with that of foragers when these are within the nest, giving them access to foraged items; (2) a substantial fraction of them (>30%) moves between the upper chambers where they were initially captured and deeper regions; and (3) their redistribution correlates and coincides with the movement of seeds and other food items downward; (4) their gradual later appearance in the role of foraging, replacing foragers that have died suggests that they are of intermediate age between brood workers and foragers; (5) their intermediate age is also supported by their intermediate fat content and location in the nest [34].

As in other tasks undertaken by social insects, the labor of transfer workers is probably organized in a series-parallel manner [1]. Thus, items as diverse as excavated sand pellets, food, leaf fragments (in leafcutter ants), trash and brood are typically transported by caching [33,35,36,37,38]. It is unlikely that any individual transport worker carries items the entire distance. The vertical mobility of transport workers, combined with their series-parallel mode of working causes them to move seeds and soil by sequential caching [33]. One must thus imagine a population of workers that constantly moves up and down over small to moderate distances within the nest performing whatever transport tasks they come upon.

The parallel patterning of labor and age in space requires that workers have information on their location. This information could conceivably be social, with ants in particular roles arranging themselves with respect to other roles. This would require cues identifying roles. Social patterning has been suggested in the tiny nests of *L*. *fasciculatus*, with workers referencing their own location relative to other groups [26]. Unspecified, but possibly social cues organized the laboratory nests of *Camponotus fellah* [28] and *Myrmica rubra* [27]. A possible source of soil depth information was proposed by Tschinkel [18] who noted a logarithmic increase in carbon dioxide concentration with depth. Ants have specific sensillae that detect carbon dioxide [39] and are known to respond to this gas with altered behavior [40,41,42]. Sadly, experimental tests showed that *P*. *badius* workers do not use soil carbon dioxide gradients as a template to arrange themselves in vertical space or to construct depth-appropriate chambers [43]. The depth cues (or social cues) used by *P*. *badius* remain unknown.

**Fig 7:**
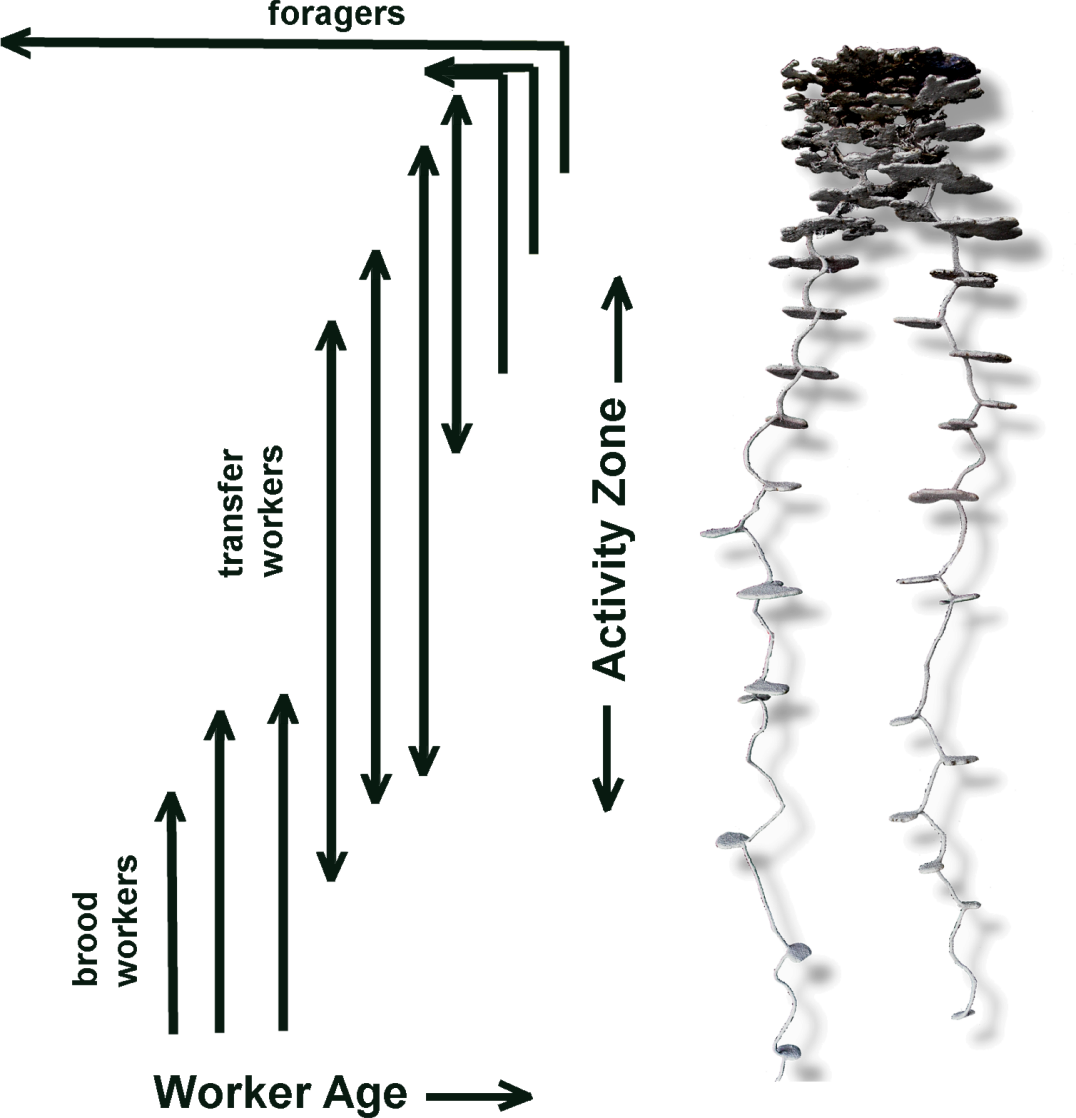
Schematic representation of the within-nest migration and movement of workers of changing roles and age. Only foragers leave the nest and die soon after doing so. They never occur deeper than about 15 cm in the nest. Transfer workers shuttle between foragers in the top of the nest and seed chambers and brood deep in the nest, shuttling materials such as food, soil and brood. As they age, they transition into sand workers and then foragers, although foragers often also dump sand, suggesting overlap between these roles.

Labor, space and age are thus all correlated, but causal links are obscure. It seems most likely that ageing results in physiological, sensory and neural changes that in turn cause workers both to change roles and location within the nest. Even in simple laboratory nests, roles are spatially segregated, as anyone who has kept pet ants can attest [27,28,44]. In *P*. *badius*, the upward movement with age is "deliberate," for when young and old workers were arranged evenly in a vertical nest, the older workers moved upward to return to the region from which they had originally been taken [18]. The upward movement is probably not a rigid movement from level to level, but one in which as workers age, they gain increased vertical mobility (in both directions) followed eventually by residence in only the upper chambers and finally foraging on the surface (Fig 7).

Our interpretation acknowledges the usually non-overlapping differentiation of labor into foraging and brood care, along with a rather vague, ill-defined, mobile and flexible transition between the two often referred to as "reserve" workers [45,46]. Our scheme is also akin to the "spatial fidelity zones" of *L*. *fasciculatus* [26], but differs from them primarily in scale and geometry—concentric millimeter-sized zones in *L*. *fasciculatus* and vertical zones stretching over meters in *P*. *badius*. Whereas the association of worker age with spatial fidelity zones was weak in *L*. *fasciculatus*, it is very strong in *P*. *badius*. Perhaps this is not surprising—the enormous dimension of the spatial fidelity zones in *P*. *badius* make the association of zone and age both necessary and obvious [16,22,34]. In both species, the spatial fidelity zones expand and contract seasonally, driven, no doubt, by seasonal demography.

Given the already apparent variation, it seems likely that the most basic worker movement is not necessarily upward in a vertical nest, as in *P*. *badius*, *Pr*. *imparis* [19] and *F*. *japonica* [21], but outward from the brood region, so that the geometry of movement differs by species and nest architectures. This links the movement in *L*. *fasciculatus* with that in the aforementioned species. In *S*. *invicta*, brood are kept in the region of optimal temperature, rather than simply the deepest part of the nest, and workers move outward to the nest perimeter and into foraging tunnels as they age [45,47]. Moreover, *S*. *invicta* moves brood rapidly in response to changes in temperature [48]. *Pheidole morrisi* also keeps brood in the warmest regions, changing locations with seasons[49].

We have thus differentiated another distinct role along the space-by-age axis. More may remain to be characterized. The distinctness or overlap among roles, and the nature and timing of transitions also remain to be elucidated. Is the shift from transfer worker to forager a gradual shift associated with experience or practice? After all, carrying out the dirt and trash requires much less skill than orienting and navigating during foraging. Perhaps transfer workers acquire the necessary foraging skills while dumping excavated sand on the surface, or doing midden and guarding work. In *Formica rufa*, inexperienced spring foragers gradually take on the characteristics of older "veteran" or "pioneer" foragers [50]. In *P*. *occidentalis*, foragers are positively phototactic and show a circadian behavioral and gene activity rhythm while brood care workers do not [51]. In *M*. *rubra*, foragers are positively phototactic while brood workers are negatively phototactic [27], a behavioral difference that may be widespread in ants. In honeybees, the transition from guard to forager is accompanied by short orientation flights. In this light, the increasingly complex behavioral capacities of ageing workers are accompanied by neural growth and increased neural complexity [52]. In principle, this question applies generally across ant species when "reserves" change roles into foragers.

Considering the ill-defined, flexible roles of reserves, these may actually be differentiated into multiple, possibly sequential roles [33,35], such as occurs in the fire ant, *Solenopsis invicta*—foragers are first recruits waiting in the nest or foraging tunnels, and then age into scouts searching for food on the surface, a role in which they live only 2–3 weeks [30]. Tschinkel et al. [33] found that some foragers of *P*. *badius* also dumped excavated sand, but non-foraging sand workers later became foragers, suggesting overlap between the two tasks. In a sense, transfer workers in *P*. *badius* may be the analogs of recruits or reserve workers, but rather than transporting items from the field into the nest, they transport items within the nest.

Demographic processes in the superorganism such as the transfer worker-to-forager transition are probably central to the efficient allocation of labor appropriate to season, colony size and other factors [22]. Our evidence that transfer workers age into foragers suggests that this is the transition shown to be sensitive to foragers mortality rate—reduction of forager mortality by penning colonies or their neighbors reduced the transition of younger workers (probably transfer workers) into foragers. Conversely, experimentally-increased forager mortality did not speed the replacement of the lost foragers [23]. The division of labor and its allocation are thus extrinsically controlled in one direction by reduced forager mortality, while the other direction is unresponsive to increased forager losses. This raises the possibility that this control echoes deep into the nest to affect the transition from brood care to transfer workers. This in turn suggests a caveat for future laboratory studies: *the lower death rate of foragers in laboratory experiments is likely to produce artifacts in the division of labor*.

Labor is a core resource for the superorganism, and like other resources such as energy and material, natural selection tends to optimize its allocation. Labor applied to one set of tasks is not available to others, so that particular patterns of allocation can be expected to maximize colony efficiency and therefore fitness. Moreover, optimal allocation may vary seasonally, with colony size and probably other factors. Our identification of transfer workers adds another element to allocation patterns already described for foragers and brood care workers within the superorganism.

## Supporting information

S1 Table Marking and recapture data. These numbers apply through initial mark-recapture of foragers, as well as through the excavation of the top 15 cm of the nest and the marking of transfer workers.
(XLSX)

S2 Table Nest excavation data. These data are from the chamber by chamber excavation of all 11 experimental colonies.
(XLSX)
